# Psychometric Properties of the Exercise Orientation Questionnaire: A Confirmatory Study on Chinese University Students

**DOI:** 10.3389/fpubh.2021.574335

**Published:** 2021-04-22

**Authors:** Jindong Chang, Liming Yong, Yali Yi, Xiaolei Liu, Hanbing Song, Yan Li, Ming Yang, Lei Yao, Naiqing Song

**Affiliations:** ^1^School of Physical Education, Southwest University, Chongqing, China; ^2^School of Mathematics and Statistics, Yulin Normal University, Guangxi, China; ^3^The Branch of the Collaborative Innovation Center of Assessment Toward Basic Education Quality, Southwest University, Chongqing, China; ^4^Key Laboratory of Physical Fitness Evaluation & Motor Function Monitoring, General Administration of Sport of China, Southwest University, Chongqing, China; ^5^Institute of Motor Quotient, Southwest University, Chongqing, China; ^6^School of Mathematics and Statistics, Southwest University, Chongqing, China; ^7^High School Affiliated to Southwest University, Chongqing, China

**Keywords:** factor analysis, reliability, psychometric, university students, exercise orientation, physical activity

## Abstract

The Exercise Orientation Questionnaire (EOQ) is a method for evaluating individuals' exercise attitudes and behaviors associated with exercise motivation. A lack of exercise motivation can affect physical activity attitudes, behavior, and action among university students. Physical inactivity may lead to health risks. The purpose of this study was to assess the measurement of psychological properties in the EOQ and to determine the reliability and validity of the EOQ when applied to Chinese university students. A total of 368 university students (male 48.8%) aged between 17 and 23 years (*M* = 19.60, *SD* = 1.18) participated in the current study. Confirmatory factor analysis (CFA) and exploratory structural equation modeling (ESEM) were used to verify the factorial validity of the EOQ. The internal consistency coefficient (Cronbach's alpha and McDonald's omega) was used to determine reliability. Multiple regression analysis was used to test concurrent validity. The International Physical Activity Questionnaire (IPAQ) was used to determine the participants' level of physical activity. The range of the subscale coefficient was 0.80–0.89, and the total scale was 0.95, which indicated that the reliability of the EOQ was excellent. The research showed that the initial CFA model of the EOQ had poorly fitting indices. The corrected model after seven residual correlations achieved the setting standard, but the correlation coefficient between some factors exceeded the standard threshold, which indicated that the CFA fitting model was not ideal. ESEM is a combination of exploratory and verifiable analytical techniques. Using ESEM and abbreviated version CFA to analyze the data indicated that the model fitted well [ESEM: TLI = 0.97 > 0.90, CFI = 0.96 > 0.90, SRMR = 0.02 < 0.08, and RMSEA = 0.045 < 0.08 (90% CI 0.033–0.055); CFA: TLI = 0.92 > 0.90, CFI = 0.91 > 0.90, SRMR = 0.08, and RMSEA = 0.06 < 0.08 (90% CI 0.055–0.067)]. The results of multiple regression analysis suggested that the ESEM model was effective in distinguishing the differences between individuals with different levels of physical activity (PAL) and body mass index (BMI). Overall, the Chinese abbreviated version of the EOQ (EOQ-CA) was fond to be a reliable tool for monitoring the exercise attitudes and behaviors of Chinese University students.

## Introduction

Unhealthy lifestyles such as low levels of physical activity, sedentary, high screen time, poor diet habits, and staying up late (short sleep duration) have become important factors affecting university students' cardiovascular disease (1–5). The World Health Organization warns individuals that physical inactivity can increase the risk of cancer, heart disease, stroke, and diabetes by 20–30% and shorten their lifespan by 3–5 years (6). Studies showed that in the United States (7), Canada (8), Germany (9), Spain (10), Portugal (11), Australia (12), Japan (13), and China (14, 15), the lack of exercise motivation was the main reason for college students' physical inactivity.

Exercise is a significant contributor to human happiness, and is of great concern to Western countries (16, 17). Individuals are motivated to engage in exercise for various reasons, such as strengthening muscles, improving skills, reducing weight, body shaping, and leisure (16, 18, 19). Previous studies showed that the motivation for physical-appearance exercise was associated with “self-control” orientation; exercising for pleasure and social reasons were related to “external-control” factors (20). The perception of exercise is a process of cognitive development from viewing it as a massive task to daily conscious actions (21). The benefits of exercise are well-known, but there are still concerns that some individuals under-exercise and others over-exercise or become addicted (19). Therefore, a tool for testing exercise orientation was needed to assess the daily exercise of ordinary individuals.

The 27-item exercise orientation questionnaire (EOQ), with 6 factors, was developed by Yates et al. (16) to observe individuals' exercise attitudes and behavior. During the development process, different patterns of motivation and perception were considered to identify obesity, eating disorders, and well-trained athletes. Their research focused on the assessment of exercise behavior to identify eating disorders or exercise addiction. Yates et al. (22) studied eating disorder (ED) risk through the evaluation of exercise attitudes and behaviors using the EOQ scale. Draeger et al. (23) studied the concept of obligatory exercise by assessing an overcommitment to exercise using the Self-Loathing Subscale (SLSS). Aruguete et al. (24) verified the reliability and validity of the SLSS as a tool for possible EDs. These studies suggest that the SLSS has high internal consistency, concurrent validity, and convergent validity. Aruguete et al. (24) also mentioned that the SLSS as a part of the EOQ was based on exercise-related issues; therefore, it was not easily identifiable by participants as a screening tool for eating disorders. Hausenblas and Downs (25) noted that overstating the similarities between eating disorders and obligatory exercisers and using unidimensional scales to assess the complete construct was not appropriate. This problem may be related to the social factors of the research object, like Yates et al.'s (26) research, which found that differences in runners, cyclists, and paddlers might be related to specific social pressures among different ethnic groups.

An assessment tool for predicting the exercise attitude and behaviors of ordinary exercisers, the EOQ, is currently the most widely used after being tested for reliability and validity. However, the EOQ scale has not been verified in China. The purpose of the current research is to examine the psychometric properties of the Chinese EOQ and determine its reliability and validity among Chinese university students.

## Materials and Methods

### Participants and Procedure

Our sample consisted of 368 university students (valid 94.1%) aged 17–23 years (*M* = 19.60, SD = 1.18). There were 48.4% male and 51.6% female participants (Table 1). The sample data were tested before the formal investigation using the Chinese Residents Exercise and Health Study (CREHS). CREHS is a national survey of Chinese residents (aged 7–65 years), including 13,787 adults and children from 34 provincial units. The CREHS aims to study the association between exercise and health in Chinese residents. The focus is on revealing the relationship between exercise habits, healthy behaviors, physical literacy, and exercise to provide analysis for public health research. The sampling method strictly adhered to the CREHS sample, which represents 95% of the total population in China.

**Table 1:** General demographic characteristics of the participants.

**Variable**	**Respondents**
Age mean (SD)	19.60 (1.18)
Height mean (SD)	168.16 (9.61)
Weight mean (SD)	59.57 (11.99)
**Gender** ***n*** **(%)**	
Male	178 (48.4%)
Female	190 (51.6%)
**PA Level** ***n*** **(%)**	
LPA	269 (73.1%)
MPA	65 (17.7%)
VPA	34 (9.2%)
**BMI** ***n*** **(%)**	
Normal	243 (66%)
Overweight	114 (31%)
Obesity	10 (2.7%)

*PA, physical activity; LPA, low physical activity; MPA, moderate physical activity; VPA, vigorous physical activity*.

Participants in the CREHS were recruited from the University Academic Group in China (CUAG). CUAG is an academic mutual aid organization. It comprises 2,000 scientific research workers from colleges and universities across the China. A member publishes survey information, and members assist each other in the questionnaire organization in their area. The preliminary investigation was conducted from 15 October to 14 November 2019 using an online questionnaire (https://www.wjx.cn/hj/k1ucgdtvduzys5szqgfxq.aspx).

A background questionnaire asked participants' gender, age, height, weight, education level, and job status. Body mass index (BMI) was calculated by using the data of self-reported height and weight. According to Chinese National Physical Health Test Standards (CNPHTS), BMI was divided into four groups: low weight (BMI < 14.8 kg/m^2^), normal (14.8 kg/m^2^ < BMI < 24 kg/m^2^), overweight (24 kg/m^2^ < BMI < 28 kg/m^2^), and obesity (BMI > 28 kg/m^2^) (27).

### Instruments

#### Instrument I: Exercise Orientation Questionnaire

This was developed to measure a range of exercise attitudes and behaviors in populations (22). It consists of six factors: self-control, exercise orientation, self-loathing, weight reduction, identity, and competition (16). The six factors with a combined total of 27 items explained 44.6% of the total variance—the alpha values of each factor ranged from 0.74 to 0.87, with the total alpha value being 0.92 (22). The concurrent validity of the EOQ was verified by the high correlation between the factor score and the regularity and intensity of exercise and self-evaluation of investment (16). A 5-point Likert scale ranging from 1 = “strongly disagree” to 5 = “strongly agree” was used inthis study (16).

The Chinese version of the EOQ was completed in three steps. First, the 27 items of the English EOQ were translated into Chinese by two authors (JC and LY). Second, two linguistics professors collectively reviewed and modified the language expression. Then, 14 students were recruited to form a focus group of 4 university students, 6 middle-school students, and 4 elementary-school students. The research team members had a face-to-face interview with them to test the experience and record the problems. Third, the research team discussed and revised the questions raised by the focus group again and finalized the Chinese version of the EOQ (28).

#### Instrument II: International Physical Activity Questionnaire (IPAQ)

This was developed by an International Consensus Group (ICG) between 1997 and 1998 (29). It was developed as an instrument that included four long and four short versions for measuring health-related physical activity in populations. For both versions, the reliability and validity of IPAQ have been extensively tested and are currently used in many international studies (30). Qu and Li (31) studied the reliability and validity of the IPAQ Chinese version and suggested that the reliability of the extended version was better than the short version. The validity of the vigorous physical activity (VPA) consistency rate was higher than moderate physical activity (MPA), and the reliability and validity of the Chinese version were consistent with the Japanese version. As IPAQ extended-version scoring was relatively complex, the ICG did not give a unified grouping standard. The data processing and analysis methods provided by Fan et al. (32) were used in this study. According to the above process, the level of physical activity was divided into three groups: VPA, MPA, and low physical activity (LPA).

### Statistical Analysis

**Figure 1:**
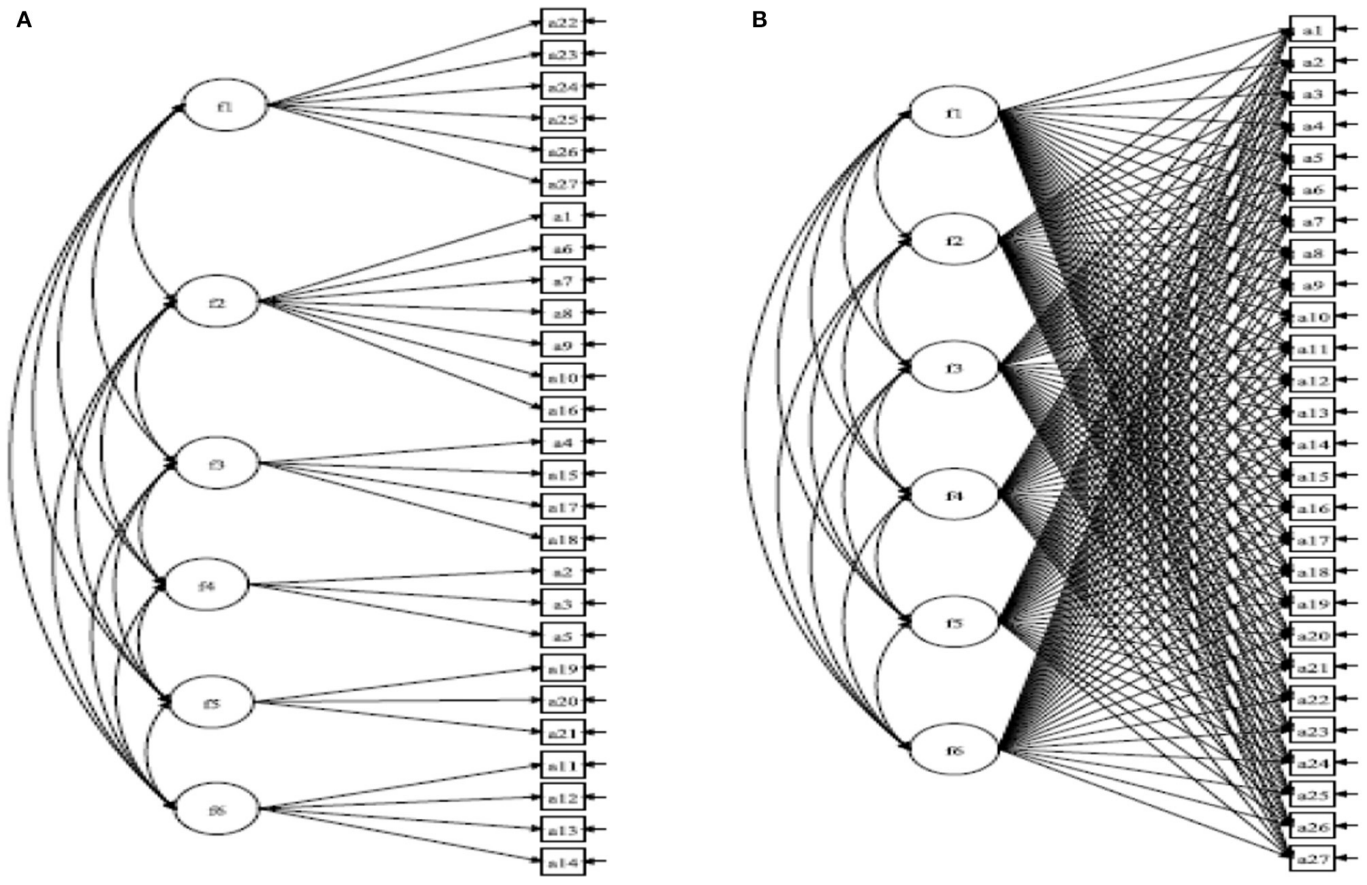
**(A)** Confirmatory factor analysis (CFA), and **(B)** exploratory structural equation (ESEM) modeling.

Statistical analysis was performed using SPSS24.0, JASP, and Mplus8.0. Descriptive statistics were derived to analyze the demographic characteristics of the sample, such as the frequency and percentage of categorical variables, and the mean and standard deviation (SD) of continuous variables. The internal consistency reliabilities of scale were judged using Cronbach's alpha and McDonald's omega coefficient. The coefficient omega (ω) and coefficient omega subscale (ω_S_) were calculated to judge the amount of variance explained by the general factor and the specific factors (33–35). Confirmatory factor analysis (CFA, see Figure 1A) and exploratory structural equation modeling (ESEM, see Figure 1B) were used to assess the psychometric properties of the EOQ by using the robust maximum likelihood estimator (MLR) (36). Hair et al. (37), evaluated model fitness against several fit indices: the comparative fit index (CFI), the Tucker–Lewis index (TLI), the root mean square error of approximation (RMSEA), and the standardized root mean square residual (SRMR). The results of CFA and ESEM were interpreted based on the following commonly used cutoff criteria for adequate model fit: χ2/df ≤ 3, CFI > 0.90, TLI > 0.90, RMSEA < 0.08, and SRMR < 0.08 (38–43). A good criterion for CFA and ESEM is that each latent variable factor should be >0.5 and ideally >0.7 (37). We conducted the test of measurement invariance of the scale's items across gender and based on published guidelines for establishing measurement invariance of models (44–46). To avoid the potential for over-fitting, we applied ESEM to conduct a mixed method of EFA and CFA to evaluate its factorial validity (47). To further verify the concurrent validity of the calibration model, we conducted multiple regression analyses in which gender, age, physical activity level (PAL) and body mass index (BMI) were measured. The regression analyses were performed with IBM SPSS Statistics (Version 24.0).

Composite reliability based on the CFA model was used to estimate the internal consistency reliability of each factor. A reliability coefficient of more than 0.70 was considered to be acceptable (48). For discriminant validity, the correlation coefficient between the two factors was lower than 0.85 as the criterion of validity (49). Based on the above fitting indicators, the applicability of the initial model was verified.

## Results

### Preliminary Analyses

Preliminary investigations showed that all items had no missing values, discrete values, or invalid values. Table 2 displayed the item correlations, means, standard deviations, skewness, and kurtosis. The correlation matrix of all items showed that only four indicators (A16 with A22; A20 with A15; A21with A15; and A21 with A5) were not statistically significant at *a* = 0.05. The preliminary analyses showed that the data were suitable for CFA. The mean score of the 27-item EOQ was 89.17 (SD = 20.63). The initial univariate skewness and kurtosis of most item scores were above the acceptable ±2.00 limit (49), indicating that the assumption of normality was not valid. Therefore, the MLR parameter estimator was considered suitable for performing CFA and ESEM (50).

**Table 2:** Correlation and description for the EOQ items.

	**A22**	**A23**	**A24**	**A25**	**A26**	**A27**	**A1**	**A6**	**A7**	**A8**	**A9**	**A10**	**A16**	**A4**	**A15**	**A17**	**A18**	**A2**	**A3**	**A5**	**A19**	**A20**	**A21**	**A11**	**A12**	**A13**	**A14**
A22	-																										
A23	0.48	-																									
A24	0.45	0.70	-																								
A25	0.39	0.64	0.60	-																							
A26	0.37	0.54	0.58	0.67	-																						
A27	0.36	0.60	0.61	0.63	0.66	-																					
A1	0.37	0.49	0.51	0.49	0.44	0.48	-																				
A6	0.37	0.48	0.49	0.47	0.49	0.49	0.56	-																			
A7	0.35	0.42	0.44	0.37	0.42	0.43	0.52	0.56	-																		
A8	0.37	0.46	0.51	0.40	0.48	0.43	0.53	0.50	0.55	-																	
A9	0.35	0.47	0.53	0.42	0.47	0.46	0.56	0.61	0.61	0.63	-																
A10	0.35	0.47	0.50	0.40	0.43	0.50	0.54	0.56	0.55	0.57	0.73	-															
A16	0.09^b^	0.29	0.35	0.29	0.34	0.29	0.27	0.35	0.21	0.32	0.30	0.29	-														
A4	0.30	0.39	0.44	0.34	0.37	0.27	0.45	0.50	0.46	0.42	0.50	0.42	0.23	-													
A15	0.18	0.24	0.23	0.17	0.11	0.13	0.26	0.27	0.21	0.28	0.23	0.12	0.22	0.44	-												
A17	0.22	0.25	0.36	0.21	0.28	0.22	0.28	0.38	0.30	0.38	0.40	0.34	0.34	0.56	0.44	-											
A18	0.32	0.45	0.53	0.38	0.43	0.35	0.41	0.47	0.41	0.49	0.49	0.48	0.36	0.52	0.37	0.61	-										
A2	0.32	0.35	0.36	0.35	0.29	0.30	0.51	0.43	0.41	0.41	0.45	0.39	0.12	0.62	0.36	0.37	0.38	-									
A3	0.32	0.46	0.49	0.48	0.37	0.44	0.51	0.49	0.36	0.39	0.49	0.42	0.22	0.51	0.27	0.38	0.43	0.58	-								
A5	0.34	0.41	0.35	0.36	0.30	0.31	0.44	0.45	0.36	0.31	0.42	0.36	0.12	0.62	0.40	0.36	0.37	0.63	0.54	-							
A19	0.44	0.50	0.57	0.41	0.49	0.47	0.46	0.49	0.50	0.55	0.55	0.55	0.37	0.26	0.10	0.27	0.46	0.26	0.34	0.26	-						
A20	0.36	0.36	0.41	0.35	0.48	0.43	0.40	0.39	0.49	0.47	0.44	0.47	0.34	0.22	0.07^b^	0.17	0.28	0.19	0.22	0.13	0.74	-					
A21	0.31	0.28	0.32	0.19	0.30	0.26	0.27	0.28	0.47	0.36	0.35	0.35	0.22	0.20	0.07^b^	0.20	0.31	0.19	0.14	0.10^b^	0.55	0.59	-				
A11	0.40	0.55	0.51	0.45	0.47	0.53	0.53	0.61	0.53	0.56	0.69	0.75	0.30	0.37	0.14	0.30	0.50	0.39	0.49	0.33	0.59	0.45	0.32	-			
A12	0.43	0.50	0.48	0.49	0.37	0.45	0.55	0.51	0.45	0.52	0.58	0.57	0.22	0.46	0.33	0.32	0.43	0.47	0.49	0.48	0.44	0.25	0.22	0.65	-		
A13	0.39	0.50	0.53	0.53	0.48	0.49	0.52	0.49	0.47	0.51	0.59	0.68	0.37	0.38	0.12	0.23	0.43	0.37	0.44	0.34	0.54	0.43	0.28	0.70	0.61	-	
A14	0.41	0.57	0.56	0.48	0.46	0.51	0.55	0.55	0.45	0.58	0.62	0.66	0.35	0.44	0.27	0.32	0.47	0.44	0.46	0.42	0.52	0.41	0.31	0.68	0.63	0.69	-
M	3.36	3.55	3.34	3.82	3.32	3.52	3.38	3.16	2.29	3.27	3.19	3.20	3.11	3.49	3.41	3.01	3.17	3.84	3.98	3.92	2.69	2.38	2.32	3.53	3.77	3.57	3.57
SD	1.15	1.12	1.16	1.01	1.21	1.09	1.26	1.18	1.21	1.21	1.24	1.19	1.10	1.30	1.06	1.29	1.18	1.20	1.08	1.23	1.13	1.13	1.15	1.15	1.06	1.04	1.06
Sk	−0.26	−0.46	−0.14	−0.56	−0.14	−0.27	−0.26	0.02	0.64	−0.13	0.08	0.08	0.09	−0.36	−0.19	0.03	0.04	−0.69	−10.03	−10.02	0.26	0.45	0.51	−0.20	−0.50	−0.01	−0.15
Ku	−0.83	−0.51	−0.88	−0.35	−0.95	−0.68	−0.93	−0.83	−0.52	−0.84	−0.103	−1.00	−0.75	−0.98	−0.70	−1.05	−0.93	−0.61	0.54	0.08	−0.69	−0.56	−0.59	−0.95	−0.55	−1.00	−0.82

b *Not a statistical association at a = 0.05*.

*sk, skewness; ku, kurtosis*.

### Internal Consistency

Table 3 listed the critical indicators of the internal consistency of the Chinese EOQ. The corrected item-total correlations (CITC) for individual items ranged from 0.34 to 0.76, indicating that most of the indicators were suitable for scale construction. The internal consistency of each subscale ranged from 0.80 to 0.89: Self-Control, 0.88; Orientation Exercise, 0.87; Self-Loathing, 0.80; Weight Reduction, 0.81; Identity, 0.83; and Competition, 0.89. The Cronbach's alpha coefficient of the Chinese EOQ was 0.95, indicating that the scale was reliable (50). Coefficient ω was high [0.95, 95% CI (0.94, 0.96)], which meant that 95% of the total variance was explained by the general factor and the specific factors. The amount of explained variance for each subscale was high, with ω_S_ ranging between 0.80 and 0.88. Thus, the internal consistency of the Chinese EOQ was acceptable (51).

**Table 3:** Item-total statistics.

**Factors/items**	**SMID**	**SVID**	**CITC**	**CAID**	**ω**	**ω_s_**	**a**
**F1: Orientation exercise**						0.88	0.88
A22	85.81	400.13	0.52	0.949	0.949		
A23	85.63	393.49	0.69	0.948	0.948		
A24	85.83	390.92	0.72	0.947	0.947		
A25	85.35	398.82	0.64	0.948	0.948		
A26	85.85	393.29	0.64	0.948	0.948		
A27	85.65	396.27	0.64	0.948	0.948		
**F2: Self-control**						0.88	0.87
A1	85.79	389.34	0.69	0.947	0.947		
A6	86.01	390.71	0.72	0.947	0.947		
A7	86.88	392.40	0.66	0.948	0.948		
A8	85.90	390.54	0.70	0.947	0.947		
A9	85.98	386.70	0.76	0.947	0.946		
A10	85.97	389.66	0.73	0.947	0.947		
A16	86.06	406.01	0.41	0.950	0.950		
**F3: Self-loathing**						0.80	0.80
A4	85.68	391.60	0.63	0.948	0.948		
A15	85.76	409.70	0.34	0.951	0.951		
A17	86.16	398.44	0.49	0.950	0.950		
A18	86.00	393.61	0.65	0.948	0.948		
**F4: Weight reduction**						0.81	0.81
A2	85.33	396.21	0.58	0.949	0.949		
A3	85.19	397.36	0.63	0.948	0.948		
A5	85.25	396.94	0.55	0.949	0.949		
**F5: Identity**						0.83	0.83
A19	86.48	393.65	0.68	0.948	0.947		
A20	86.79	399.55	0.54	0.949	0.949		
A21	86.85	404.43	0.43	0.950	0.950		
**F6: Competition**						0.88	0.89
A11	85.64	390.33	0.75	0.947	0.947		
A12	85.40	394.99	0.69	0.948	0.948		
A13	85.60	395.28	0.71	0.948	0.947		
A14	85.60	392.96	0.75	0.947	0.947		
Total						0.95^#^	0.95^#^

*SMID, scale mean if item deleted; SVID, scale variance if item deleted; CITC, corrected item-total correlation; CAID, Cronbach's alpha if item deleted*.

# *Cronbach's Alpha of full scale*.

*ω, coefficient omega; ω_S_, coefficient omega subscale; a, Cronbach's alpha*.

### Factorial Validity

**Figure 2:**
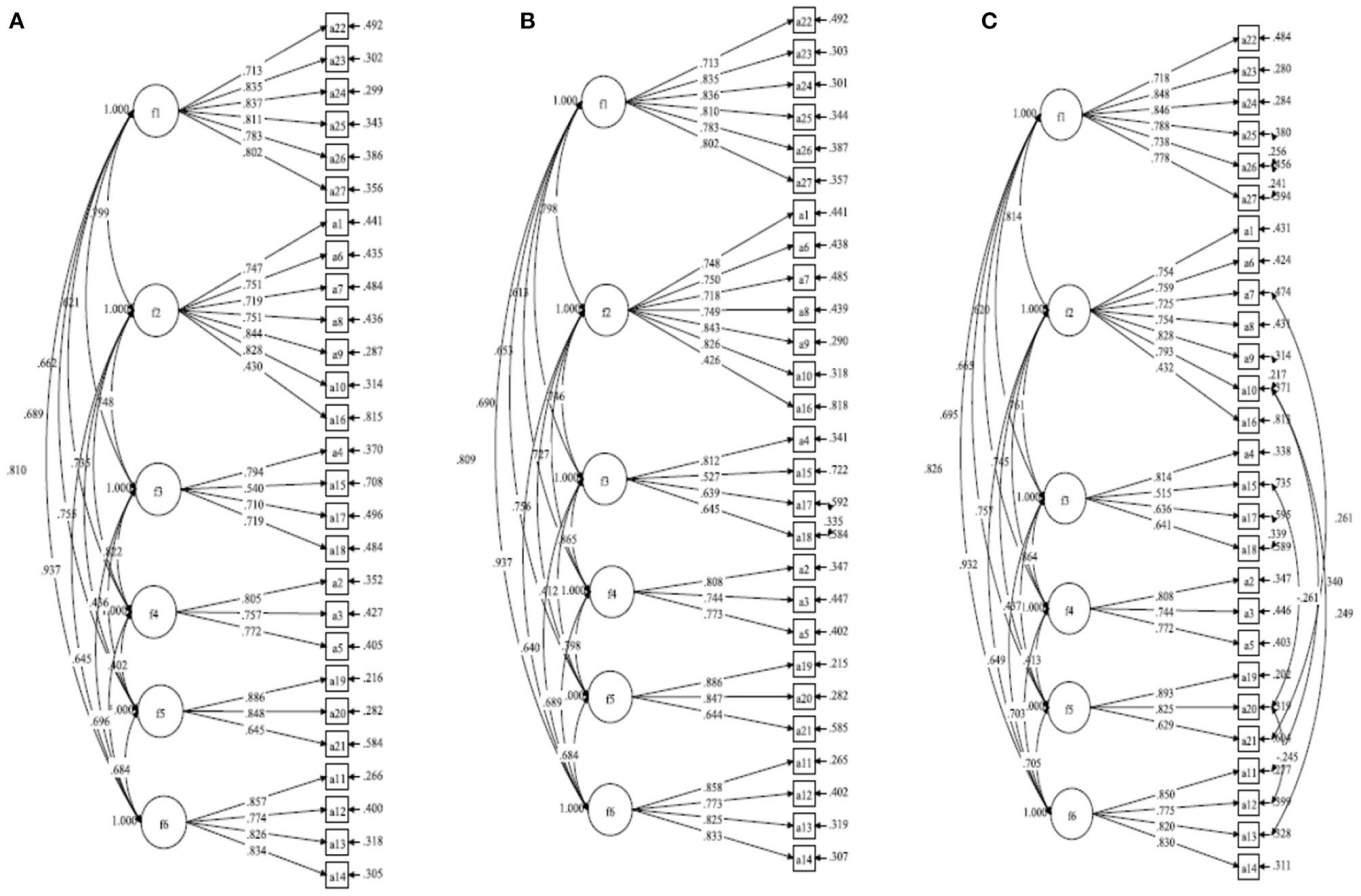
Standardized factor loading for **(A)** Model-1, **(B)** Model-2, and **(C)** Model-8.

The CFA results of the initial measurement model (Model-1) reported poor factorial validity. The EOQ that included six factors with a 27-item structure failed to meet most of the criteria for a good model fit, with χ^2^/df = 810.077/315 = 2.57 < 3, *p* < 0.001, TLI = 0.897, CFI = 0.885, SRMR = 0.084, and RMSEA = 0.065 (90% CI 0.06–0.071). Although the loading of all items was >0.40 (see Figure 2A), the poorly fitting indices indicated that Model-1 did not fit the data well (52). For models with inadequate fit, it has become common practice to modify the model by deleting unimportant parameters and adding parameters that can improve the fit (49).

Next, we modified the initial measurement model according to the model parameter adjustment principle (49). Based on Model-1, the residuals for item A18 and A17 were correlated to establish a modified Model-2 (Figure 2B). The fitting results of Model-2 showed that the value of χ^2^ decreased by 24.57, CFI increased by 0.05, TLI increased by 0.15, RMSEA decreased by 0.01, and SRMR decreased by 0.01. Although each fitting index of the model was improved to a certain extent (see Table 3), the fit indices of TLI and SRMR were outside the recommended values. The factor loadings of all items in Model-2 were above 0.40 (Figure 2B).

According to the same model modification principle, the residuals of items A11 with A10, A20 with A15, A20 with A12, A21 with A7, A26 with A25, and A26 with A27 were correlated in turn, and Model-3, Model-4, Model-5, Model-6, Model-7, and Model-8 were established simultaneously (see Table 4). Finally, all fitting indices of Model-8 were within the recommended values (see Figure 2C). However, there was a higher correlation between f2 and f6 with *r* = 0.932 (*p* = 0.015), and f3 and f4 with *r* = 0.864 (*p* = 0.038). Two pairs of correlation r-values exceeded 0.85, indicating that the discriminant validity had some degree of misfit with the model.

**Table 4:** Model fit indices for nine Exercise Orientation Questionnaire (EOQ) models.

**Model**	**χ^2^**	***df***	**AIC**	**BIC**	**CFI**	**TLI**	**SRMR**	**RMSEA (90% CI)**	**Modification index**
Model-1	810.077	315	25546.077	25897.804	0.897	0.885	0.084	0.065 (0.060, 0.071)	Initial model
Model-2	785.507	314	25517.968	25873.604	0.902	0.890	0.083	0.064 (0.058, 0.069)	A18 with A17
Model-3	761.002	313	25490.120	25849.664	0.907	0.896	0.085	0.062 (0.057, 0.068)	A11 with A10
Model-4	741.245	312	25469.184	25832.636	0.911	0.900	0.083	0.061 (0.055, 0.067)	A20 with A15
Model-5	727.671	311	25456.263	25823.623	0.913	0.902	0.083	0.060 (0.055, 0.066)	A20 with A12
Model-6	709.590	310	25435.831	25807.099	0.917	0.906	0.082	0.059 (0.053, 0.065)	A21 with A7
Model-7	696.074	309	25418.511	25793.687	0.919	0.909	0.081	0.058 (0.053, 0.064)	A26 with A25
Model-8	682.227	308	25401.004	25780.088	0.922	0.911	0.080	0.057 (0.052, 0.063)	A26 with A27
Model-9	334.017	204	25145.032	25930.556	0.973	0.953	0.020	0.042 (0.033, 0.050)	ESEM-6F

Further investigation was conducted using ESEM to explore the fitting validity of the model. The fit of the ESEM model (Model-9) showed that the data were acceptable on the fit indices, with TLI = 0.973, CFI = 0.960, SRMR = 0.020, and RMSEA = 0.045 (90% CI 0.33–0.55) (see Table 3). The chi-square test of model fitness was significant, χ^2^/*df* = 334.017/204 = 1.64 < 3, *p* < 0.001, indicating some degree of misfit with the model. Compared with Model-8, the correlation coefficient between each factor of Model-9 established using ESEM was <0.85 (see Table 5), indicating that the fitting of the ESEM model was better than the CFA model (27-item).

**Table 5:** Confirmatory factor analysis and exploratory structural equation modeling standardized factor correlations based on the EOQ.

	**CFA (Model-8)**					**ESEM (Model-9)**					**CFA (Model-R3)**				
	**F1**	**F2**	**F3**	**F4**	**F5**	**F1**	**F2**	**F3**	**F4**	**F5**	**F1**	**F2**	**F3**	**F4**	**F5**
F2	0.81					0.49					0.79				
F3	0.62	0.76				0.21	0.63				0.65	0.78			
F4	0.67	0.75	0.86^*^			0.42	0.67	0.57			0.65	0.73	0.88^*^		
F5	0.70	0.76	0.44	0.41		0.42	0.42	0.32	0.32		0.69	0.75	0.46	0.40	
F6	0.83	0.93^*^	0.65	0.70	0.71	0.22	0.17	−0.05	0.25	0.07	0.81	0.94^*^	0.67	0.69	0.68

*F1, self-control; F2, orientation exercise; F3, self-loathing; F4, weight reduction; F5, identity; F6, competition*.

* *Correlation coefficient r > 0.85*.

**Figure 3:**
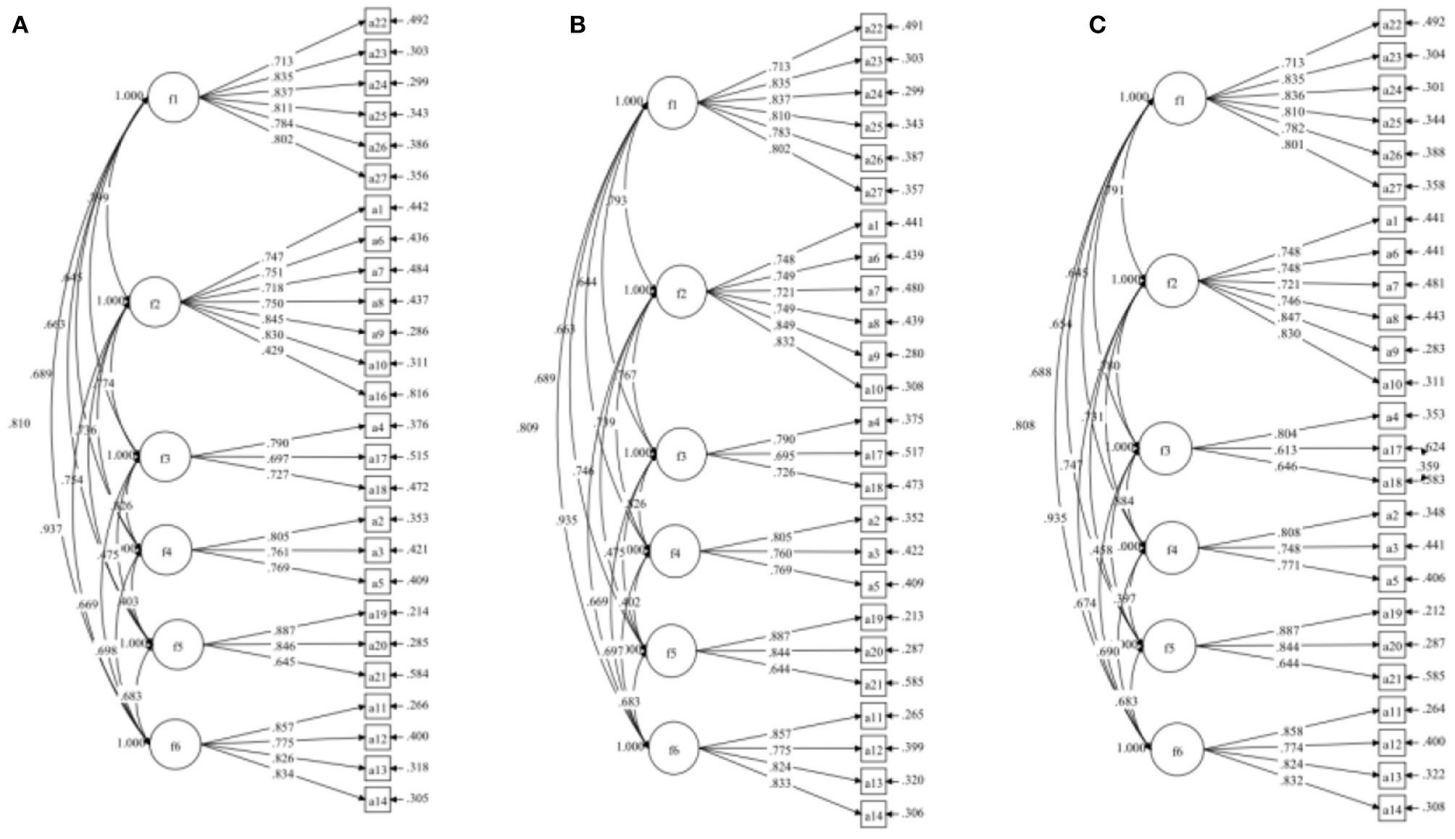
Standardized factor loading for **(A)** Model-R1, **(B)** Model-R2, and **(C)** Model-R3.

Table 3 presented an analysis Cronbach's alpha if item deleted (CAID). The results showed that the removal of an item did not have a significant impact on the value of the Cronbach's alpha. One of the potential solutions is to shortened the scale or create an abbreviated version (53–56). Therefore, we tried to shorten the scale for the CFA version. Based on the CITC and model fitting index, item A15 was deleted to build a new modified Model-R1. In the same way, Model-R2 was built by deleting item A16. The modified Model-R2 approximate fitting index was >0.90, and the correlation load between each item and factor was >0.6. According to Hermida's recommendations (57), we considered the relevant correction of item residuals for Model-R2. When the residuals of A17 and A18 were related, Model-R3 met the fitting standard (see Table 6 and Figure 3).

**Table 6:** Model fit indices for abbreviated EOQ models.

**Model**	**χ^2^**	***df***	**AIC**	**BIC**	**CFI**	**TLI**	**SRMR**	**RMSEA (90% CI)**	**Modification index**
Model-1	810.077	315	25546.077	25897.804	0.897	0.885	0.084	0.065 (0.060, 0.071)	Initial model
Model-R1	727.548	290	24553.474	24893.477	0.905	0.894	0.083	0.064 (0.058, 0.070)	Delete A15
Model-R2	657.653	266	23496.738	23825.017	0.913	0.902	0.083	0.063 (0.057, 0.069)	Delete A16
Model-R3	629.316	265	23463.700	23795.887	0.919	0.908	0.080	0.061 (0.055, 0.067)	A17 with A18

The Model-R2 as baseline model (configural model) was used to test measurement invariance for boys and girls. The configural invariance model fit the data well (see Table 7), since all three fit criteria (CFI, TLI and RMSEA) indicated good model fit. The first constrained mode (Model-R4), the weak invariance model, fit the data well (see Table 7). Changes of CFI, TLI, and RMSEA, when the weak invariance model was compared with the configural model, were within recommended values (ΔCFI = −0.003, ΔTLI = 0, ΔRMSEA = 0). This indicated that the items used to estimate the factor loadings had the same meaning for males and females. The second constrained model (Model-R5), the strong invariance model also fit the data well (see Table 7). The results showed the strong invariance (ΔCFI = −0.005, ΔTLI = 0.002, ΔRMSEA = 0.001). The last constrained model (Model-R6), which constrained the factor loadings, item intercept, and residual variances, to produce the strict invariance model was then inspected. The changes of the fit indices were within the acceptable values (ΔCFI = −0.003, ΔTLI = 0.001, ΔRMSEA = 0). Taken together, it can be concluded that the measurement invariance across gender was valid.

**Table 7:** The tests of measurement invariance for abbreviated EOQ models.

**Model**	**χ^2^*(df)***	**CFI**	**TLI**	**RMSEA**	**Model comparison**	**Δχ^2^*(df)***	**ΔCFI**	**ΔTLI**	**ΔRMSEA**
Model-R2 (configural)	939.921 (920)	0.916	0.903	0.066	-	-	-	-	-
Model-R4 (weak)	973.544 (539)	0.913	0.903	0.066	R4 vs. R2	33.623 (19)	−0.003	0	0
Model-R5 (strong)	995.293 (558)	0.913	0.906	0.065	R5 vs. R4	21.749 (19)	0	0.003	−0.001
Model-R6 (strict)	1034.800 (583)	0.910	0.907	0.065	R6 vs. R5	39.508 (25)	−0.003	0.001	0

Table 8 listed multiple regression coefficients describing the effect of participants' gender, age, PAL, and BMI on the factor scores of the Chinese abbreviated version of the EOQ (EOQ-CA). The results showed that gender factor had a significant impact on EOQ-CA (*p* < 0.05) and its subscale Orientation Exercise (*p* < 0.05) and Identity (*p* < 0.05). Self-Loathing (*p* < 0.05) and Identity (*p* < 0.05) were affected significantly by the age factor. PAL and BMI had a significant impact on EOQ-CA and each of its subscales (*p* < 0.01). Further analysis of the PAL subgroup showed the mean of all factors differed significantly (*p* < 0.01), with the exception of Self-Loathing (*p* < 0.05) (see Table 9). In addition, the mean results of different groups found that VPA subgroup scored the highest in all factors, followed by MPA, and LPA was the lowest (see Table 9).

**Table 8:** Standardized multiple regression coefficients (Partialβ) of Model-R3.

	**Gender**		**Age**		**PAL**		**BMI**	
	**β**	**95% CI**	**β**	**95% CI**	**β**	**95% CI**	**β**	**95% CI**
SC	−0.071	(−1.836, 0.330)	−0.067	(−0.763, 0.156)	0.194^**^	(0.756, 2.448)	0.339^**^	(2.422, 4.329)
OE	−0.112^*^	(−2.480, −0.148)	−0.081	(−0.896, 0.093)	0.247^**^	(1.330, 3.152)	0.275^**^	(1.953, 4.052)
SL	−0.078	(−1.142, 0.150)	−0.104^*^	(−0.554, −0.006)	0.156^**^	(0.264, 1.273)	0.186^**^	(0.505, 1.695)
WR	−0.026	(−0.766, 0.450)	−0.108^*^	(−0.532, −0.016)	0.192^**^	(0.414, 1.364)	0.294^**^	(1.100, 2.190)
I	−0.053	(−0.919, 0.293)	0.039	(−0.160, 0.354)	0.148^**^	(0.204, 1.151)	0.175^**^	(0.408, 1.528)
C	−0.131^*^	(−1.715, −0.230)	−0.122^*^	(−0.699, −0.070)	0.218^**^	(0.676, 1.835)	0.276^**^	(1.245, 2.577)
EOQ	−0.101^*^	(−7.954, −0.060)	−0.092	(−3.221, 0.128)	0.243^**^	(4.349, 10.516)	0.325^**^	(8.515, 15.489)

* *p < 0.05*;

** *p < 0.01*.

*SC, self-control; OE, orientation exercise; SL, self-loathing; WR, weight reduction; I, identity; C, competition; PAL, physical activity level; BMI, body mass index*.

**Table 9:** Descriptive of mean scores of different PAL and BMI values.

**Group**	**Subgroup**	**SC**	**OE**	**SL**	**WR**	**I**	**C**	**EOQ**
PAL	LPA (*n* = 255)	20.28 ± 5.07	17.61 ± 5.51	9.38 ± 3.05	11.43 ± 3.00	7.13 ± 2.88	13.96 ± 3.54	79.78 ± 18.40
	MPA (*n* = 54)	22.37 ± 5.17	20.35 ± 5.95	10.32 ± 3.41	12.32 ± 2.91	7.91 ± 2.98	15.40 ± 3.86	88.68 ± 20.20
	VPA (*n* = 26)	23.15 ± 6.58	21.85 ± 6.43	10.76 ± 3.30	13.09 ± 2.55	8.56 ± 3.21	16.38 ± 3.92	93.79 ± 23.22
	F	7.59	12.74	4.60	6.27	4.82	9.46	11.92
	P	0.001^**^	0.000^**^	0.011^*^	0.002^**^	0.009^**^	0.000^**^	0.000^**^
BMI	Obesity (*n* = 10)	16.30 ± 4.62	15.30 ± 2.83	9.10 ± 2.56	9.80 ± 2.57	6.50 ± 2.42	12.80 ± 2.39	69.80 ± 7.81
	Overweight (*n* = 114)	18.41 ± 3.92	15.95 ± 3.62	8.66 ± 2.81	10.46 ± 3.03	6.56 ± 2.47	12.79 ± 2.88	72.82 ± 12.24
	Normal (*n* = 243)	22.30 ± 5.41	19.82 ± 6.34	10.19 ± 2.76	12.43 ± 2.76	7.83 ± 3.10	15.30 ± 3.83	87.87 ± 20.95
	F	28.06	20.50	9.66	21.07	7.90	20.78	28.35
	P	000^**^	000^**^	000^**^	000^**^	000^**^	000^**^	000^**^

* *p < 0.05*;

** *p < 0.01*.

*SC, self-control; OE, orientation exercise; SL, self-loathing; WR, weight reduction; I, identity; C, competition; PAL, physical activity level; BMI, body mass index*.

Additionally, Tables 8, 9 displayed the relationship between the EOQ-CA and its subscales with the measures related to with BMI. One-way ANOVA in the BMI group found that the EOQ-CA and its subscales were significant different between obesity, overweight, and normal university students' group (*p* < 0.01). Table 9 showed the normal subgroup scored the highest in all factors, followed by the overweight subgroup, and the obesity subgroup was the lowest. Thus, the results demonstrated that the EOQ-CA using the translated abbreviation had good concurrent validity.

## Discussion

The intention of developing the EOQ was to evaluate the exercise attitudes and behaviors of college students, including their psychological characteristics (16). The purpose of this study was to determine the psychometric characteristics of applying the EOQ to Chinese college students. The confirmatory factor analysis of 27 items showed that the initial model did not achieve model fitness. Accordingly, a model re-specification was performed based on high modification indices by correlating the residual items (58). The fitting parameters of the final model (Model-8) after seven corrections were acceptable. However, the discriminant validity of the modified model—the correlation threshold between the two factors was more than 0.85—did not meet the set standard. After further exploration and verification of the data using the ESEM model (Model-9) and abbreviated version model (Model-R1, R2, R3), the fitting indices of the Model-9 and Model-R3 met the required standard. Multiple regression analysis suggested that gender and age had a partly significant effect on EOQ scores, whereas physical activity level (PAL) and body mass index (BMI) had a significant effect. A comparison of the mean EOQ scores showed that the higher the physical activity level, the higher the EOQ score, and the difference was significant among different physical activity levels. Similarly, normal-weight college students scored higher than the overweight and obesity groups, and the difference was significant.

The factor structure of the initial EOQ was constructed using EFA, and its concurrent validity was verified by the correlation between exercise and EOQ score (16). Although the initial scale reported good reliability and validity, the Chinese version still required a confirmatory analysis considering the cross-cultural differences. The results when using the CFA model to verify the Chinese version of the factor structure suggested that the fit indices of the initial model did not meet the fitting criterion. Re-specification was conducted based on the initial measurement model using seven residual correlations; the model fit indices achieved the basic fitting standard, but the discrimination validity of the CFA modified model was inadequate (49).

Subsequently, ESEM and abbreviated CFA were used to explore the fit indices of the model, and various indicators showed that the ESEM model and abbreviated model (EOQ-CA) fitted better than the original CFA model (48). Multiple regression analysis showed that the level of physical activity and BMI affected the EOQ score, and the higher the level, the greater the score (50). Interestingly, age did not appear to be related to the EOQ score. This confirmed that the age of the initial scale was not significantly correlated with the EOQ score (22). In the initial scale, F3 (Self-Loathing) was the only factor that did not establish concurrent validity. However, the concurrent validity of Self-Loathing was confirmed in this study (22).

As reported in numerous review articles, CFA models often fail to meet standards of good measurement because of the strict requirement of zero cross-loadings (59–62). This overly restrictive assumption results in “biased parameter estimates which permeate across other parameter estimates in the model” (63). There is another possible explanation to explain why the CFA results did not fulfill the criteria for model fit: because the original developers of the scale only relied on EFA for its development, the scale may suffer from a potential problem of factorial validity. This problem is common for those scales developed before CFA became popular and user friendly. One of the potential solutions is to shorten the scale or create an abbreviated version (53–56). We shortened the scale by deleting two items and verified that the concurrent validity of EOQ-CA showed a greater fit. Measurement invariance of the abbreviated version questionaries also supports the consistency of evaluations between males and females. ESEM is an optimal integration between EFA and CFA that incorporates many advantages of CFA, but its limitation is a lack of freedom (47). ESEM has been widely used in psychological research but, to date, no study has applied ESEM to the EOQ (61, 64–66).

In summary, the ESEM model and CFA abbreviated version model have better fitting parameters than the original CFA model (63). The reliability and validity of the EOQ-CA fit the required standard, and it is a reliable tool for measuring college students' exercise attitudes and behavior.

Nonetheless, the present study still has some limitations. The first relates to data management. Self-managed reporting methods may cause bias in the collected data. Second, there was a lack of professional college athletes among the participants. In future research, first, we will strengthen the design to address these issues and increase the diversity of research objects. Second, we will enlarge the sample size and monitor the quality of sample data. In addition, we will carry out EOQ-CA measurement invariance test and introduce a two-factor model to further verify the structure of EOQ-CA.

## Conclusions

The 25-item Chinese abbreviated version of the EOQ (EOQ-CA) scale, with six factors, provided an acceptable model fit for good scale reliability. The results of the present study show that the EOQ-CA can be used to predict exercise attitudes or behavior of Chinese University students in relation to physical activity and BMI. The verification of the EOQ-CA scale also expands the global study and application of this assessment instrument.

## Data Availability Statement

The raw data supporting the conclusions of this article will be made available by the authors, without undue reservation.

## Ethics Statement

The studies involving human participants were reviewed and approved by The Scientific Ethics Committee of Southwest University. Written informed consent to participate in this study was provided by the participants' legal guardian/next of kin.

## Author Contributions

JC, LiY, YY, XL, YL, HS, MY, and LeY: data collection. JC, LiY, YY, and NS: data analysis, conception, and design. JC, YY, and NS: research design, writing the manuscript and revision. All authors contributed to the article and approved the submitted version.

## Conflict of Interest

The authors declare that the research was conducted in the absence of any commercial or financial relationships that could be construed as a potential conflict of interest.
